# Tax obsessions: Taxpayer registration and the “informal sector” in sub‐Saharan Africa

**DOI:** 10.1111/dpr.12649

**Published:** 2022-11-29

**Authors:** Mick Moore

**Affiliations:** ^1^ International Centre for Tax and Development UK; ^2^ Institute of Development Studies UK

**Keywords:** Africa, informal sector, politics, tax, tax administration

## Abstract

**Motivation:**

There are three puzzling features of sub‐Saharan African tax systems: tax administrations maintain records on vast numbers of small enterprises that actually provide no revenue; they continually invest resources into registering even more of these “unproductive taxpayers”; and discussions about taxing small enterprises are framed by the ambiguous, misleading concept of the “informal sector.”

**Purpose:**

To make sense of these separate puzzling practices and narratives by exploring the synergies between them, and the broader organizational and political interests that they serve.

**Methods and approach:**

There is little statistical or sociological information on the functioning of national tax administrations in sub‐Saharan Africa. The analysis is based on the results of recent research; along with a thorough search for useful data; my own extensive interactions with African tax administrators and relevant international organizations; and a sensitivity to the political dimensions of taxation.

**Findings:**

The three features of tax systems that are individually puzzling make sense when examined holistically. The continual drive to register more taxpayers provides an unduly favourable impression of the extent of policy and managerial efforts to collect more revenue. The informal sector narrative locates the apparent cause of revenue scarcity in the alleged undertaxation of small enterprises and poorer people, and thus helps divert attention from failures adequately to tax more privileged Africans and larger enterprises.

**Policy implications:**

Be very wary of claims that it would be a good idea to invest resources in registering large numbers of new taxpayers in sub‐Saharan Africa. Try to avoid using the term “informal sector” when discussing issues of tax policy and administration—it is confusing and diversionary.

## 1 INTRODUCTION

This article addresses three related questions:

- Why do national tax administrations in sub‐Saharan Africa typically maintain such very large banks of “unproductive taxpayers,” i.e. individuals and businesses who are registered for tax purposes, may or may not submit tax returns, but fail to actually provide any revenue?
- Why, despite these large banks of unproductive taxpayers, do these same national tax administrations suffer from what I term the “registration obsession,” i.e. a tendency to devote considerable resources to registering even more taxpayers—who similarly will provide little additional revenue?
- Why does the notion of an undertaxed informal sector appear so frequently in discussions of tax in the region, despite the ambiguity of the term and its irrelevance to the practical steps needed to improve the quality, coverage, and efficiency of tax administration?

These seem rather esoteric questions. They are likely only to engage people already immersed in issues of tax policy and administration in Africa. They are also novel questions. One would not come across either the questions themselves or the facts on which they are based in the standard literature on tax policy and administration, on Africa or elsewhere. The hardest and most precise facts, notably statistics on the prevalence of unproductive taxpayers, derive from very recent research conducted with African tax administrations, using their internal data. Much of that research has been done by my colleagues in the International Centre for Tax and Development, in collaboration with staff from the eSwatini, Rwanda and Uganda Revenue Authorities. This article leans heavily on that research (see Section 3) and a range of other statistics obtained after a thorough trawl of all available research and official reports on tax administration in contemporary Africa. The origins of the article lie in my puzzlement when I first saw the evidence that the same national tax administrations were simultaneously trying to register more and more taxpayers even when the majority of taxpayers already on their books were not actually generating any revenue. The phrase “registration obsession” and the first version of the arguments presented here appeared in an earlier working paper (Moore, 2020). The current article is based on rather more data and some deeper reflection.

And the answers to the three questions above? In sum, I suggest that sub‐Saharan African tax administrations waste resources by maintaining large banks of unproductive taxpayers and by continually seek to expand those banks through new registrations because this fits the narrative about the undertaxation of the so‐called “informal sector.” The behaviour and the narrative collectively work to the advantage of more privileged groups. They help the people who run African national tax administrations and their political superiors to appear to be working harder than they actually are to raise more revenue. And the undertaxed rich and wealthy benefit from a narrative—and actions consistent with that narrative—that imply that it is the poor in the informal sector who are failing to pay their fair share of tax. However scholars may define the informal sector, when the term is used in relation to tax in Africa, it is dominantly understood to refer to poor people and small‐scale economic operators.

Section 2 deals with the question of who, from the perspective of a tax administration, should be considered a (direct) taxpayer. More precisely, to grasp the realities of tax administration and the scope for raising additional revenue, we need to understand the limitations of the binary distinction, made so frequently in general discussions of taxation, between (registered) taxpayers and non‐taxpayers. We need a larger number of categories, including “unproductive taxpayers”—individuals or enterprises that are registered for tax purposes but do not actually pay taxes. In Section 3, I explain the sources of the statistics used in this article, and then deploy them to show that unproductive taxpayers are surprisingly numerous in sub‐Saharan Africa. The reasons for their prevalence are discussed in Section 4. A major reason is the registration obsession—a focus on adding new taxpayers to the register despite a wide range of evidence that the great majority will remain unproductive. Possible reasons for the registration obsession are examined in Section 5. An important motivation is the informal sector narrative—the concept of a seriously undertaxed informal sector, which should be a priority target for additional revenue raising. I suggest in Section 6 that this informal sector narrative is conceptually confused and serves political purposes, notably pinning the blame for inadequate revenue collection on poorer people. The disappointing experiences of trying to raise additional revenue from small‐scale enterprises in sub‐Saharan Africa are summarized in Section 7. The policy implications are in Section 8.

## 2 WHO IS A TAXPAYER?

The term *taxpayer* is commonly used to mean different things. There is, first, a distinction between (a) those entities that directly remit revenue to a government agency, and (b) those that consciously or unconsciously actually bear some of the economic burden of paying the cost of indirect taxes, like sales and excise taxes, customs duties, and value added tax (VAT). The concern here is with a less familiar distinction between: (a) entities that actually remit taxes; and (b) others that do not actually remit taxes but are considered “taxpayers” from an organizational or legal perspective because they are registered with a tax collection authority. As will become evident below, the difference between the two categories can be numerically and organizationally significant.

We can better understand the reasons for this gap by looking in more detail at tax administration procedures. There are several steps in the process between being registered as a taxpayer and remitting taxes. The number of steps depends on the way in which tax collection is organized. This may vary from one country to another, and from one tax to another within any country. For present purposes, we can focus on the processes for collecting the three main revenue sources of most governments in the world, including sub‐Saharan Africa: VAT, corporate income tax (CIT) and personal income tax (PIT).^1^ In order to collect revenue through any of these three taxes, a tax administration needs to take potentially taxable entities through the following basic stages (Moore, 2020, p. 21):

- First, they need to be registered. If they remain unregistered, they should have no substantive dealings with the tax collector.
- Once registered they are normally required to file—to submit annual (or quarterly or monthly) tax returns with information on their income, turnover, costs, and profits, etc.^2^ If registered taxpayers fail to submit tax returns, they are labelled “non‐filers.”
- Although they file, their returns may indicate either no economic activity (no income, no profit), or such a small amount of activity that they fall below the threshold of tax liability. In either case they are then labelled “nil‐filers.”
- “Positive filers”—registered taxpayers who are neither non‐filers nor nil‐filers—receive an assessment and a tax bill. They might fail to remit, and thus become “non‐payers.”
- Only registered taxpayers who file positive returns and pay what is due become taxpayers in the strictest sense of the term.

There is then no unique or correct operational definition of the word taxpayer. The same is true of the term “unproductive taxpayers.” Generically, an unproductive taxpayer is a taxable entity that is registered for tax, but not actually paying it. We could define it as comprising all non‐filers, nil‐filers, and non‐payers. Purely for pragmatic reasons—because data on non‐payers is rare for sub‐Saharan Africa^3^—the operational definition of unproductive taxpayers used here is non‐filers plus nil‐filers. But that does not exhaust the definitional questions. For how many consecutive years should a registered taxable entity fail to file before it merits the label non‐filer? And similarly for nil‐filers? There is no obvious answer, no standard practice, and a scarcity of reliable incidence data of any kind. In Section 3, I use what reliable data is available, and accept the definitions used by the people who generated that data.

## 3 HOW MANY UNPRODUCTIVE TAXPAYERS ARE THERE IN SUB‐SAHARAN AFRICA?

It is impossible to give a definitive answer to this question. The only way of obtaining the required information is to have research access to what is termed “tax administration data”—the data on taxable entities that revenue administrations compile and keep in the course of collecting taxes.^4^ With the partial exception of South Africa, virtually no research of this nature had been undertaken in sub‐Saharan Africa five years ago. There is now a small amount, on which this article draws. I scoured all plausible sources, and came up with information on six countries:

- In their submission to the International Survey on Revenue Administration (ISORA) for 2016, the Nigerian federal revenue authorities declared the following proportions of unproductive taxpayers: 98% for PIT, 94% for CIT, and 95% for VAT (IMF, 2018, p. 7).
- The Malawi Revenue Authority defines as unproductive those taxpayers who are registered but have filed no tax return and/or made no tax payment over a three‐year period. In the 2015–2016 fiscal year, over 50% of taxpayers were unproductive (Ligomeka, 2019, p. 14).
- Mascagni and Mengistu (2016) examined all annual CIT returns filed with the Ethiopian Revenues and Customs Authority over an eight‐year period (2006/7–2013/14). They had no access to statistics on non‐filers. Among the firms who filed, around half were nil‐filers.^5^
- The only known attempts to carefully measure the incidence of unproductive taxpayers in sub‐Saharan Africa have been made recently in eSwatini, Rwanda, and Uganda (separately for CIT and PIT). The detailed figures are in Moore (2020, p. 22). The estimates of unproductive taxpayers as a proportion of all taxpayers range from 60%–90%, and average 73%.

Unproductive taxpayers are not just making occasional appearances on taxpayer registers in sub‐Saharan Africa. If the six countries cited above are representative of the region, then on average considerably more than half the taxpayers registered with national tax administrations are not paying taxes at all.^6^ We do not know that the countries are representative. None are Lusophone, and only Rwanda is Francophone—albeit in many respects now part of the Anglosphere. However, there is evidence from another source that is at least consistent with the suggestion that the phenomenon of unproductive taxpayers is widespread, and probably even the norm. In recent professional appraisals of the performance of tax administration functions in sub‐Saharan Africa, national tax administrations on average have received especially low scores for the quality of their taxpayer registers—the basic information they maintain on potentially taxable entities.^7^ This would not be the case if the great majority of registry entries were being used regularly, and most taxpayers were actually generating revenue every year. There would then be more incentive to maintain accurate registers.

It is unclear how far this bloating of taxpayer registers with unused and often inaccurate data on unproductive taxpayers undermines the efficiency of tax administrations. It surely makes them less effective in terms of day‐to‐day operations. Further, the potential to undertake analysis of the data for management purposes—one of the main potential benefits of digitalization for tax administrations—is severely diminished.

The case for not having so many unproductive taxpayers on registers seems clear. Why are they there? It is possible that this phenomenon is also widespread in other parts of the world. There is insufficient evidence on that. But it is clear that it is uncommon in Organisation for Economic Co‐operation and Development (OECD) countries.^8^ We need to explain why the situation is so different in sub‐Saharan Africa.

## 4 WHY ARE THERE SO MANY UNPRODUCTIVE TAXPAYERS?

The immediate reason for the large number of unproductive taxpayers is evident. In at least five of the six countries on which data is given in the previous section, there had been recent campaigns to increase the number of registered taxpayers.^9^ It is virtually inevitable that the great majority of any consequent new registrations will be of small or very small businesses. It seems highly unlikely that any African tax administration would permit large numbers of medium or large businesses to remain unregistered. The cost of revenue foregone is too large.

Some of the more striking statistics come from Nigeria. As stated in Section 3, in 2016 the Nigerian federal revenue authorities reported the following proportions of registered taxpayers to be unproductive: 98% for PIT, 94% for CIT, and 95% for VAT. These figures referred to the situation after “successful initiatives” to register new taxpayers, which led to 530,000 new corporate registrations in the first quarter of 2016—a 67% increase (IMF, 2018, p. 7). One would not expect all these newly registered taxpayers to be paying much revenue immediately after registration. Let us assume that none of them paid any revenue in 2016. The implication, from the figures given in the source, is that, for CIT, 88% of existing registered taxpayers were unproductive before the launch of a campaign to register more.

The Uganda statistics are equally vivid. When staff of the Uganda Revenue Authority (URA) investigated the condition of their taxpayer register in 2018, they found a very large number of registration errors. It was difficult to identify or contact many registered taxpayers (Mayega et al., 2019, p. 13). At that time, more than half the registered taxpayers had not communicated or engaged with the URA for at least two years—and far fewer had actually paid any tax (Mayega et al., 2019, p. 13). A major reason for this situation was the formal success of a previous large‐scale, sustained campaign to register new taxpayers, involving several government agencies.^10^ Over the period 2009 to 2017, the number of registered taxpayers grew from less than 20,000 to 1.3 million—increasing by a factor of 70 (Mayega et al., 2019, p. 9). The online registration procedure was flawed, easily generated mistakes, and incorporated little verification (Mayega et al., 2019, pp. 11–15). This suggests that registration targets took precedence over the practical usefulness of the registrations for revenue collection.

In fiscal year 2015/16, the Malawi Revenue Authority classified over half of registered taxpayers as unproductive—defined as not having filed a tax return or made a tax payment within the past three years. One‐third (35%) of all registered taxpayers were registered for what is termed the “turnover tax regime.” This is one of many variants of presumptive or simplified tax regimes widely used for small enterprises in many parts of the world (Cleary et al., 2017, p. 29). No attempt is made to examine business accounts or estimate profits. Rather, tax assessments are based on a simplified indicator of taxability (Bird, 2004; Bird & Wallace, 2003; Thuronyi, 1996). The fact that a high proportion of Malawian taxpayers were registered for the turnover tax regime was the outcome of a project, initiated in 2010, to register as many small taxpayers as possible. Temporary staff had been hired to identify and register potential candidates. In 2015/16, the 35% of taxpayers registered under the turnover tax regime accounted for just 1% of total tax revenue collection. Even so, the Revenue Authority was continuing to seek out additional candidates and register them for turnover tax (Ligomeka, 2019, p. 14).

It would be useful to know more about the extent of pressure and incentives to register new taxpayers in more normal times, outside registration campaigns. But that information would not change the conclusion that the existence of a large proportion of unproductive taxpayers is, at least in significant part, the result of explicit decisions to use scarce resources to increase registrations.^11^ Note further that, if a tax administration is expending resources on registering taxpayers who are unlikely to supply any revenue, it will almost certainly simultaneously be registering taxpayers who will be unproductive in a weaker sense of the term: the revenue they provide will not cover the costs incurred by the tax administration in engaging with them (see Section 7).

## 5 WHY THE OBSESSION WITH REGISTRATION?

Why are tax administrations in Africa so keen on bulking up their taxpayer registers? There are several possible explanations.^12^

First, there are valid technical reasons for tax administrations to collect tax from some small enterprises, even if they thereby incur costs rather than generate revenue. This procedure may help identify and socialize into compliance small and new enterprises that will eventually grow and become significant revenue providers. It might discourage larger enterprises from slipping through the tax net by legally presenting themselves as several separate companies each too small to tax. And, in the right hands, it can generate information about trends in business structures and practices that are useful to the tax administration. But these objectives justify the maintenance of only a small stock of registered unproductive taxpayers, not the large numbers that we actually observe.

Second, the registration obsession might reflect less visible institutional and political dynamics. Heads of governments and tax administrations, like leaders of almost all public organizations in contemporary states, are under constant pressure to solve problems, or to appear to be doing so—a phenomenon sometimes labelled “performative governance” (Brunsson, 1989; Ding, 2020). Registering additional taxpayers is not a daunting task for a tax administration—it already has the organizational machinery available. It can report achievements in simple numerical terms. Field‐level tax collectors can report success to their superiors. Tax administration leaders can report to their ministers. Ministers can report to international organizations and aid donors. Success in meeting new registration targets can almost be guaranteed in advance.^13^

We have some direct evidence that the registration obsession has been encouraged from outside professional tax administration circles. Aid donors have sometimes used new registrations as a performance indicator for their revenue projects in Africa (Moore, 2020, p. 25). Less tangibly, external agencies also exercise an adverse effect through a more discursive channel. The International Monetary Fund (IMF), World Bank, and other international organizations continually advise African governments to “broaden the tax base.” This is intended in part to be a diplomatic way of saying “stop giving unjustified tax exemptions and ensure that those who can make a substantial contribution actually do so.” But the messaging is oblique. The recipients may take the words at face value: “get more people to pay tax, and start by getting them onto the tax register.” However, neither aid donors nor other external agencies have so much influence over national tax administration in Africa that they can be considered the primary sources of the registration obsession.^14^

While the two possible explanations for the registration obsession listed above are both plausible, they are unlikely to account for the intensity or pervasiveness of the registration obsession. There are two further potential explanations, which lie even more in the realm of ideas and ideology. My argument is based on observations of the degree to which this belief in the importance of increasing the number of registered taxpayers goes hand in hand with a way of thinking about the revenue problem in Africa that I label the informal sector narrative. The essence of my interpretation of the role of the informal sector narrative is as follows:

- The question of inadequate government revenue is frequently and unhelpfully framed as a problem of inability to adequately tax the informal sector (or informal economy).
- This is unhelpful, first, because the term informal sector is ambiguous. The people who use the term in reference to taxation are not agreeing on anything substantive. They are simply using the same phrase.
- The term is unhelpful, second, because there is a clearer, more useful, and more standard way of labelling those economic activities that are not captured in official economic statistics—shadow (or underground, parallel, grey, black, etc.) economy.
- It is unhelpful, third, because the informal sector framing implies that a prime cause of inadequate tax collection is the failure of smaller businesses (and poorer people) to pay tax. It distracts attention from the actual major potential sources of additional revenue, including the incomes and assets of wealthier people.
- Taken together, the informal sector narrative and registration obsession are diversionary at the level of policy and practice. They provide both a (misleading) definition of the problem and an (unproductive) strategy for solving it—registering lots of small enterprises.

I expand on this summary interpretation in Section 6.

## 6 THE INFORMAL SECTOR NARRATIVE

Official and unofficial conversations and publications about tax in Africa are replete with references to the informal sector, and to its alleged role as a source of tax evasion.^15^ This is curious. Why would people seeking to identify sources of significant uncollected revenue need to refer to the informal sector at all? On the one hand, there is a better, standard term that is used globally: “tax gaps.” On the other hand, the term informal sector is extremely imprecise. In essence, definitions of the concept oscillate between two poles. One is the notion of unrecorded economic activity–activity unknown to government agencies that does not then feature in basic economic statistics. There is a term for this: the shadow (or underground, parallel, grey, black, etc. economy) (OECD, 2017). Enormous efforts are put into measuring the size of shadow economies. Measurement is very challenging. Unlike the informal sector, the core concept of the shadow economy is clear. The other definitional pole of the informal sector is the notion of small‐scale economic activity, encompassing small‐scale enterprise and the irregular, flexible, and unrecorded relationships between small‐scale businesses and the people who work for them. The ambiguity of the concept is on display in, for example, the definition of the informal sector in Wikipedia (“Informal economy,” 2022).^16^

Why then is such an ambiguous term as the informal sector used so widely with reference to the revenue problem in sub‐Saharan Africa, especially when standard global practice is to employ more precise terms like the shadow economy and the tax gap? I can think of two possible explanations. The first stems from the general human bias towards wanting to explain things, rather than admit ignorance and uncertainty (Lent, 2017, p. 30), and specifically from the strong psychological and political pressure on people in political authority to adhere to a limited range of policy narratives that imply that they have a good understanding of the policy problems they face and viable solutions for them (Roe, 1991). It is actually very difficult to calculate tax gaps for most countries in sub‐Saharan Africa.^17^ The relevant statistics and the capacity to do the basic analysis are scarce. Equally scarce in almost all tax administrations is the investigative capacity needed to move, for example, from an estimate that 50% of VAT due is not collected, to a strategy to identify the major defaulters and the networks of inter‐business transactions in which they are embedded.^18^ So the narrative that closely associates missing revenue with the informal sector might thrive in part because policy‐makers do not have available a more convenient or comforting interpretation of the problems they face.

But that interpretation is not only speculative, it is also deficient: it does not take into account the reality that, in practice, the informal sector narrative is used extensively to pin the blame for missing revenue on small‐scale enterprises. To simplify, it is a way of pointing to an alleged need to collect more tax from small‐scale enterprise—and thus from many poor people—without admitting that they may be poor. One piece of evidence comes from a recent large‐scale study of the problem of taxing the informal sector published by the African Tax Administration Forum, the main professional organization for senior tax administrators in Africa. The study opens with a discussion of the “numerous and diverse definitions” of the informal sector, and then plumps for a definition that is itself expansive, but places the emphasis on activities that escape government records.^19^ However, the later listing of the key characteristics of the informal sector—non‐payment of taxes, low productivity, non‐registered entities, temporary working premises/structures, located in busy markets, individual or family ownership (African Tax Administration Forum, 2021)—makes it crystal clear that the focus is on taxing small‐scale enterprise. If their initial definition of the informal sector as unrecorded economic activity were actually used, the study would have taken account of large‐scale unrecorded gambling and financing, cigarette manufacturers colluding with smugglers to avoid excise duties (Gallien, 2020), or doctors and other well‐paid professionals accepting only cash payments. A key practical indicator of the unrecorded informal economy from the tax collectors' perspective is large‐scale cash transactions, not small family businesses operating from temporary premises. Note also that in contemporary sub‐Saharan Africa a popular tool for trying to identify and register the informal sector for tax is the Block Management System. In essence, this involves dividing urban areas into small blocks, and sending teams of tax collectors to identify, and if necessary register, all people engaging in remunerative economic activity in each block. This may be a good way to identify people who are selling meals on the street, or providing portering services to the retail trade. It is not a good way to detect unofficial banking or other large‐scale unrecorded economic activity. If the latter is the objective, the principal reason for identifying unrecorded small‐scale operators is not to tax them, but to use them to map networks and then track down the kingpins—and tax them.

There is complementary evidence about the actual interpretation put on the notion of the informal sector in tax collection practices. Most tax administrations in the world tend to focus their assessment and collection resources on larger taxpayers, typically through a Large Taxpayer Unit focused on companies, and another unit focused on wealthy people (High Net Worth Individuals) (OECD, 2019). The latter is where the money is. Most African tax administrations now have a Large Taxpayer Unit (Moore, 2020, p. 14), but few have a unit focusing on wealthy individuals. In a recent survey of 26 national tax administrations for the *2018 African Tax Outlook*, only four had “special sections for High Net Worth Individuals (HNWIs).” By contrast, 15 reported one or more “special programs or initiatives to deal with the informal sector” (African Tax Administration Forum, 2018, p. 107).

## 7 THE EXPERIENCE OF TAXING SMALL ENTERPRISE IN AFRICA

Leaving aside conceptual and definitional issues, does it make sense for national revenue authorities in sub‐Saharan Africa to try to raise more revenue by focusing on small‐scale businesses? Of course, many small enterprises do not pay the tax they are formally required to pay. That is true globally.^20^ But, even leaving aside issues of equity, there are major questions, familiar to tax administrators, about the low potential cost–benefit ratio of focusing organizational resources on small‐scale business.

The costs of collection are likely to be high. Most of the revenue collected may be eaten up in collection costs. With the significant exception of the results of Rachel Beach's excellent ethnographic research with tax collectors in Benin and Togo, there seems to be almost no publicly available data on this issue for the Africa region (Beach, 2018, especially chapter 8). There are, however, good reasons to believe that the benefits, in terms of additional revenue, from concentrating tax administration resources on small‐scale business will tend to be unusually low in sub‐Saharan Africa. The reason is that, in many African economies, small numbers of large firms typically account for a high proportion of economic activity, and thus of total tax collections. The only comprehensive data we have on revenue remitted by firm size comes from Rwanda. The Rwanda Revenue Authority classifies all taxpayers as large, medium, or small and micro. In 2019–2020, just 375 large taxpayers, accounting for 0.06% of all registered taxpayers, remitted 58% of all tax revenue. 843 medium taxpayers (0.36% of the total) remitted 12% of revenue, and the 99 + % labelled small and micro taxpayers remitted 30% of revenue. The average large taxpayer remitted USD 2.3 million, while the average small and micro taxpayer remitted USD 1800 (Rwanda Revenue Authority, 2020, pp. 34–35).^21^ These numbers vividly illustrate just how challenging it is to significantly increase tax revenue in Africa by focusing on small taxpayers. For example, the Rwandan Revenue Authority could increase total revenue by 5% through expanding the number of small and micro taxpayers by 32% (73,000 units) and ensuring that each new recruit remitted half of the average amount of money collected from all taxpayers in this category—USD 900. Alternatively, it could achieve the same total increase in revenue by collecting just 9% extra from each of the 375 large taxpayers.

In light of these realities, it is not surprising that the limited evidence available suggests that the experience of trying to collect additional revenue directly from small‐scale economic activities has been disappointing. These initiatives often involve the presumptive or simplified tax regimes mentioned in Section 4, that in principle obviate the need to calculate income. A recent review of these presumptive regimes in four African countries concluded: “The evidence suggests that the revenue potential from informal sector taxation is low, in part because of the difficulty in designing and administering effective informal sector tax regimes” (Dube & Casale, 2016, p. 601).^22^ Another recent careful piece of research reaches a similar conclusion. In 2008, and again in 2014, the South African Revenue Service (SARS) used its high information technology (IT) capacity to expand the number of businesses registered for business tax by simply importing all the details of companies listed in the commercial registry held by the Companies and Intellectual Property Commission. On both occasions, the number of companies registered with SARS for business tax increased by about 10%. Although SARS is generally a high‐performing organization, there was very little impact on the amount of revenue raised (Lediga et al., 2020).

It is worth dwelling briefly on a recent research publication that claims a more positive result. It is based on an analysis of two simultaneous and inter‐related programmes in Uganda: one to register new taxpayers and the other to encourage them to pay a presumptive tax. In their summary, the researchers claim that “presumptive tax revenues increased substantially,” and that the benefits of the registration programme in terms of revenue collected “outweigh the costs” (Jouste et al., 2021). However, the main text tells a different story. Presumptive tax revenues did increase, but from a miniscule base. At the end of the programme, they accounted for only 0.04% of total revenue. And even that figure is based on the assumption that all tax declared as payable by taxpayers in their tax returns was successfully collected (Jouste et al., 2021). The only cost taken into consideration in doing the cost–benefit analysis was the direct cost of the registration programme to the URA itself. No account was taken of (1) costs incurred by other agencies involved in the registration programme; (2) the compliance costs incurred by the newly‐recruited taxpayers; and above all, (3) the additional routine costs to the URA of processing more tax returns, auditing, billing, and collecting (Jouste et al., 2021). Towards the end of the article, the authors change tack. They state that the main objective of the programme “was to formalize businesses – not to increase revenue collection” (Jouste et al., 2021). In this context, “formalizing businesses” means nothing more or less than registering them with the Revenue Authority. There is no explanation of why these additional registrations should be seen as positive. The taxpayer registration drive under examination is the one mentioned in Section 4, through which the total number of taxpayers registered with the URA increased by a factor of 70 over a nine‐year period. At the point the research was done, the URA already had a very high proportion of unproductive taxpayers on its books (see Section 3). The message seems to be that a tax administration cannot go wrong in registering new taxpayers. Even if it does not contribute to the goal of raising more revenue, it contributes to the objective of registering more taxpayers. Someone will be impressed.

## 8 CONCLUSIONS

This article draws attention to three practices or narratives around tax administration in sub‐Saharan Africa that are widespread and, taken separately, somewhat puzzling:

- National tax administrations maintain very large banks of unproductive taxpayers—individuals and businesses who are registered for tax purposes, may or may not submit tax returns, but fail to actually provide any revenue.
- Despite these large banks of unproductive taxpayers, these same national tax administrations often devote considerable resources to registering even more taxpayers—who similarly will provide little additional revenue.
- The notion of an undertaxed informal sector appears frequently in discussions of tax in the region, despite the ambiguity of the term and its irrelevance to the practical steps needed to improve the quality, coverage, and efficiency of tax administration.

I suggest there are synergies between these three phenomena. Behaviours that appear irrational in isolation make sense when examined collectively. I believe that these phenomena collectively work to the advantage of two networks of relatively privileged actors:

- The first are the people who run national tax administrations and their political superiors—and occasionally the international organizations that aid and advise them. They can escape some of the responsibility for their failure in recent decades to increase the proportion of gross domestic product collected as taxes (Gupta & Liu, 2020). By focusing ostentatiously on increasing the number of registered taxpayers, they give a misleading impresssion of the degree to which they are trying to tackle the deeper and much more political problem of actually collecting more revenue.
- The second are privileged Africans more generally. They share and help replicate an interpretation of the revenue situation that they find comforting and is in their interest: there is a revenue deficit; much of the cause lies in the ability of small‐scale enterprises (the informal sector) to avoid paying taxes; and their governments and tax collectors are actively trying to solve that problem by registering small enterprises for tax.

This triad, comprising unproductive taxpayers, the registration obsession, and the informal sector narrative, reduces the efficiency and effectiveness of national tax administrations by focusing attention and energy on unimportant, non‐strategic issues and measures. More importantly, it helps divert attention from the inequities of most African national tax systems, and the failure of the privileged to pay their fair share.^23^ Although the interpretations here of the political and ideational dimensions of this triad are provisional and subject to the findings of future research, two strong policy conclusions emerge:

- Be very wary of claims that it would be a good idea to invest resources in registering large numbers of new taxpayers.
- Try to avoid using the term “informal sector” when discussing issues of tax policy and administration in sub‐Saharan Africa. It is confusing and diversionary.

## FUNDING INFORMATION

The article was funded with grants from the Bill and Melinda Gates Foundation (OPP1197757) and the Foreign, Commonwealth and Development Office (300211–101).

## Data Availability

The data that support the findings of this study are available from the corresponding author upon reasonable request.

## Footnotes

^1^
A different process is involved for the collection of annual residential property taxes. This would typically require: the initial entry of the property into the property tax register; occasional revaluations, typically every 10 or more years; the issuing of annual tax bills; and enforcement of payment. Similarly, excise taxes, customs duties, and withholding taxes are each associated with a distinct process.

^2^
Alternatively, and especially in cases where PIT is deducted from salary by the employer and paid directly to the tax administration, the filing of the relevant information is effectively done by a third party.

^3^
For example, in 2016 in Nigeria it was not possible from official records to determine the proportion of taxpayers who actually made payments in response to assessments. It was “generally believed to vary between 15 and 40 per cent for VAT” (IMF, 2018, p. 7).

^4^
That tax administration data should also be sufficiently accurate. This is not always the case (Mayega et al., 2019). Among the evidence listed here, the strongest comes from research in eSwatini, Rwanda, and Uganda that involved considerable efforts to verify and clean tax administration data (Mascagni et al., 2020; Santoro & Mdluli, 2019).

^5^
23% of firms had reported zero economic activity or revenue for every year in which they filed returns; in an average year about a third of returns indicated revenue lower than costs, and thus no CIT liability.

^6^
The main potential sources of more consistent and accurate information on the incidence of inactive taxpayers in Africa do not help. The International Survey on Revenue Administration has not been very successful in attempts to collect accurate data on the ratio of active taxpayers to registered taxpayers (Crandall et al., 2019, pp. 63–64). The statistics on active taxpayers (as a percentage of registered taxpayers) for 22 African countries in Table 5.3 of the *African Tax Outlook 2019* are seriously misleading (African Tax Administration Forum, 2019). Overall, the table indicates that, for VAT, CIT, and PIT, active taxpayers on average represented 76%, 62%, and 69% of registered taxpayers respectively. But several countries, including Rwanda, reported figures of 100% for each of the three taxes. The eSwatini figures were close to 100%. In total, 53% of entries to the table were for 90% or above. The experience of the researchers who did the work reported in Table 1, which shows much lower figures, suggests that a major reason is probably that many tax administrations do not know the true figures, and cannot easily determine them because of the poor state of their tax registers and other records.

^7^
The reference is to the appraisals undertaken under the aegis of the International Monetary Fund (IMF) using the Tax Administration Diagnostic Assessment Tool (TADAT, n.d.). Access to the results of these assessments for 28 countries in sub‐Saharan Africa was kindly granted by the TADAT Secretariat. The data that supports this conclusion about the particularly poor quality of taxpayer registers is in Moore (2020, p. 20).

^8^
There is no exactly comparable data available for OECD countries. In 2019, the proportion of registered taxpayers who filed their returns on time for four major taxes—PIT, CIT, VAT, and Pay As You Earn (PAYE)—ranged between 80% and 88% for all OECD countries reporting. The figures for on‐time payments ranged from 83% to 91% (OECD, 2021, pp. 67, 70).

^9^
The Nigeria, Uganda, and Malawi cases are discussed in the main text below. Santoro and Mdluli (2019, p. 9) provide evidence on registration campaigns for eSwatini, and Mascagni et al. (2020, p. 8) do so for Rwanda.

^10^
Most of this expansion resulted from the Taxpayer Registration Expansion Project, which was targeted at the informal sector. It was a joint operation between the Uganda Revenue Authority, the Uganda Registration Services Bureau, the Kampala Capital City Authority, and other sub‐national governments, such that registration with any one agency resulted in automatic registration with the others. Large numbers of government staff were employed to make physical checks on potential business enterprises on a locality basis.

^11^
At any point, the existence of some unproductive taxpayers is inevitable for technical reasons. In particular, there will always be a few legitimate nil‐filers—business owners who happen to be making losses or tiny profits in any tax period, especially at start‐up, but who intend to continue in business and become profitable.

^12^
The analysis in this and the succeeding section is to some degree speculative. The speculation is, however, based on extensive interactions with African tax collectors and policy‐makers over more than a decade, notably in the period 2010–2020 when I was chief executive officer of the International Centre for Tax and Development, and travelling to Africa frequently for formal meetings and for advisory work.

^13^
To the extent that the registration obsession reflects a need for tangible evidence of apparent effort to increase tax collection, it is probably initiated more by politicians and government leaders than senior tax administrators. The latter generally know better. Investing organizational resources in registering large numbers of unproductive taxpayers runs contrary to the normal instincts of senior staff in tax administrations and to the incentives they face. It absorbs resources, rather than generating revenue. In sub‐Saharan Africa, the dominant performance indicator for tax administrations—the one that their ministers of finance and heads of government take seriously—is meeting annual revenue collection targets.

^14^
And it is only for a small minority of donor tax projects that I have evidence of the use of new registrations as a key performance indicator.

^15^
My perception derives principally from listening to many conversations about taxation within African government, elite, and middle‐class circles. Inadequate revenue and the undertaxed informal sector are also linked in a mountain of written material of all kinds, including scholarly articles, comments, blogs, and speeches. An online search on Google under “tax Africa informal sector” on 18 November 2020 generated almost 28 million references. The terms “informal sector” and “informal economy” appear 35 times in a recent flagship report from the United Nations Economic Commission for Africa titled “Fiscal Policy for Financing Sustainable Development in Africa” (UNECA, 2019).

^16^
This ambiguity reflects history. The term “informal sector” was invented to refer to conditions in low‐income Africa in the middle of the twentieth century, when employment in large‐scale enterprises was rare and—unlike today—other types of economic activity, and indeed much of the population, was largely undocumented, and illegible from the official perspective (Gerxhani, 2004). The scholarly publications that show up in a Google Scholar search for the term informal sector predominantly focus on small‐scale economic activities.

^17^
This is evident in a recent analysis of the tax gap in Ghana (World Bank, 2020).

^18^
The South African Revenue Service is probably the only significant exception to this generalization.

^19^
The actual wording is: “For the purpose of the analysis herein, this guidebook considers the informal sector to include all economic activities by workers and economic entities which are not covered or insufficiently covered by formal arrangements including underground production activities and not registered by RAs (revenue authorities) or TAs (tax authorities)” (African Tax Administration Forum, 2021). Note that there seems to be redundancy involved in debating the meaning of the term informal sector in relation to tax deficits, only to end up with a definition that simply covers potential taxpayers not registered with tax authorities.

^20^
Some of the most reliable estimates of national tax gaps are produced by the UK national revenue authority. Their estimate for the 2019/20 financial year was that 50% of the tax gap was attributable to small businesses and (non‐wealthy) individuals, and the other 50% to medium and large businesses, criminals, and wealthy individuals (HM Revenue and Customs, 2022).

^21^
The figures are also available for the previous year, and are nearly identical (Rwanda Revenue Authority, 2019, p. 33). There are other incomplete sources of information that point to similar patterns, including figures for VAT only for eight African countries in 2004–2009 (Kloeden, 2011, p. 26), and figures that exclude excise and customs revenues for 12 African countries for 2014 (African Tax Administration Forum, 2016, p. 56).

^22^
It is likely that one reason for the poor performance of these special regimes is that they differ from standard tax regimes on paper more than in practice. Under normal regimes, tax collectors seem to frequently make assessments on the basis of what they believe individual enterprises can or should pay, and then file paperwork that suggests that assessments were based on calculation of actual profit (Beach, 2018, chapter 8). The obstacles to effectively and economically taxing small businesses in Africa through central national tax administrations are severe (Moore, 2020), with or without presumptive or other special regimes. For example, many small‐scale businesses do not operate continuously or predictably. Operations may cease for a wide range of reasons, many of them unpredictable. They include seasonal factors, illness, the emergence of other temporary livelihood opportunities, and unavailability of working capital. The existence of a presumptive tax regime does not reduce the problems that the taxpayer faces in getting exemption from taxes during these periods when the business is not operational, or trying to claim refunds for taxes already paid.

^23^
Because of the absence or poor quality of much of the data needed to assess the degree of equity in African tax systems, this claim cannot be substantiated in detail. The evidence, however, strongly suggests that this is the case (Moore et al., 2018, chapter 7; Kangave et al., 2016; Kangave et al., 2018; Kangave et al., 2020).
